# Wild-type oestrogen receptor beta (ER*β*1) mRNA and protein expression in Tamoxifen-treated post-menopausal breast cancers

**DOI:** 10.1038/sj.bjc.6602183

**Published:** 2004-10-12

**Authors:** P A O'Neill, M P A Davies, A M Shaaban, H Innes, A Torevell, D R Sibson, C S Foster

**Affiliations:** 1 Clatterbridge Cancer Research Trust, J.K. Douglas Laboratories, Clatterbridge Hospital, Bebington, Wirral, CH63 4JY, UK; 2 Department of Cellular and Molecular Pathology, University of Liverpool, L69 3GA, UK

**Keywords:** breast cancer, ER gene expression, tamoxifen, oestrogen receptor beta, tumour progression

## Abstract

This study has tested the hypothesis that comparison of protein and mRNA expression for ER*α* and ER*β*1 by human breast cancers provides novel information relating to the clinical and pathological characteristics of human breast cancers. Expression of ER*α* and ER*β*1 was identified in 167 invasive cancers from postmenopausal women treated only with endocrine therapy. The cohort included 143 cases receiving only adjuvant Tamoxifen following surgery. ER*α* and ER*β*1 expression was analysed by immunohistochemistry and reverse transcription RT–PCR and compared with clinical progression of individual cancers. ER*α* protein was closely associated with the corresponding RNA detected by RT–PCR (Chi-square, *P*<0.001). In contrast, ER*β*1 protein and mRNA were inconsistent. Although an association was identified between ER*α* and ER*β* mRNAs (Chi-square, *P*<0.001) and between ER*α* protein and ER*β*1 mRNA (Chi-square, *P*<0.027), no association was identified for the ER*α* and ER*β*1 proteins detected by immunohistochemistry. ER*β*1 was not associated with outcome. However, in the absence of ER*α*, ER*β*1 protein expression was associated with elevated cell proliferation. There was a trend for the ER*β*1 protein-positive cases to have a worse outcome, both within the group as a whole as well as within the ER*α*-positive Tamoxifen-treated cases. This study has confirmed the hypothesis that expression of ER*α* is an important determinant of breast cancer progression, and has further demonstrated that ER*β*1 may play a role in the response of breast cancers to endocrine therapy.

Currently, ER*α* expression is regarded as a reliable prognostic marker with which to predict the response of an individual breast cancer to hormone therapy (Pertschuk and Axiotis, 1999). However, up to 40% of breast tumours with positive ER*α* status do not respond to endocrine manipulation (Locker, 1998). The biological basis of this failure to respond is poorly understood, although modulated expression of ER*β* has been implicated. Unlike ER*α*, the antioestrogen–ER*β* complex inhibits gene transcription when bound to oestrogen response elements (EREs), but acts as an agonist when bound to AP1 elements (Paech *et al*, 1997). Therefore, it is possible that antioestrogens may have agonistic effects in ER*β*-positive breast tumours, resulting in a lack of efficacy of hormonal therapy. This hypothesis is supported by a small number of cases in which overexpression of ER*β* RNA has been found in Tamoxifen-resistant tumours (*n*=9) when compared with a Tamoxifen-sensitive group (*n*=8) (Speirs *et al*, 1999), but refuted by an immunohistochemical study (Mann *et al*, 2001) of ER*β* protein in a larger group (*n*=118). Hitherto, only a limited number of studies have used ER*β*-specific antibodies (Jarvinen *et al*, 2000; Omoto *et al*, 2001; Saunders *et al*, 2002). These have been based on relatively small numbers of unselected cases and do not all address the relationship of ER*β*1 expression with patient outcome. Similarly, there are limited studies addressing the specific relationship of ER*β* with endocrine therapy (Speirs *et al*, 1999; Mann *et al*, 2001; Saji *et al*, 2002). Hence, the present study has been restricted to an assessment of postmenopausal women receiving endocrine therapy, but no chemotherapy, in order to better address the likely impact of ER*β*1 expression on response in this common clinical setting.

Recent development of reliable antibodies to ER*β*, as well as to ER*α*, has allowed examination of the protein expression of these genes (Taylor and Al-Azzawi, 2000; Choi *et al*, 2001; Mann *et al*, 2001; Miyoshi *et al*, 2001; Omoto *et al*, 2001; Roger *et al*, 2001; Skliris *et al*, 2001, 2002; Saji *et al*, 2002; Saunders *et al*, 2002). One previous report suggested a lack of correlation between mRNA and protein for total ER*β* in 37 out of 61 tumours studied (Shaw *et al*, 2002). Consequently, there is a lack of available data on the possible significance of ER*β* expression in specific treatment cohorts. Furthermore, many previous studies have not adequately defined the precise ER*β* variants being measured or have used antibodies capable of detecting multiple variants (Skliris *et al*, 2003). The relatively high levels of ER*β*1 protein identified in positive cases may indicate that the PPG5/10 antibody, employed in this study, is among the most sensitive presently available for use in immunohistochemistry (Skliris *et al*, 2002) with the protocol employed herein (Shaaban *et al*, 2003a). Using this antibody, we have already confirmed that the level of ER*β*1 detected in normal breast epithelium and in premalignant breast lesions is greater than formerly recognised (Shaaban *et al*, 2003a, 2003b). Therefore, the purpose of this study was to test the hypothesis that comparison of protein expression levels of ER*α* and ER*β*1, together with their respective mRNA levels, in a cohort of postmenopausal primary breast cancer patients treated with surgery and hormonal therapy, would accurately predict the clinical and pathological characteristics of these cancers.

## MATERIALS AND METHODS

### Patients

Patients undergoing treatment for invasive breast cancer during the period 1993–1999 were identified within the archival database of the Department of Pathology at the Royal Liverpool University Hospital, and the Cancer Tissue Bank Research Centre (CTBRC) in the same institution. The study population comprised a group of 167 postmenopausal women treated with surgery either with, or without, radiation treatment (Table 1

**Table 1:** Histological, clinical and molecular characteristics of 167 breast cancer cases receiving adjuvant endocrinetreatment but no chemotherapy

**Characteristic**	**Group**	***n***	**%**
Histology	Invasive ductal	141	84
*n*=*167*	Invasive lobular	13	8
	Other	13	8
Surgery	Wide local excision	104	62
*n*=*167*	Mastectomy	63	38
			
Endocrine therapy	Tamoxifen adjuvant only	143	86
	Other adjuvant only	7	4
*n*=*167*	Primary and adjuvant	17	10
Radiotherapy	No	89	53
*n*=*167*	Yes	78	47
Stage	I	38	23
*n*=*167*	II	93	56
	III	7	4
			
Grade	I	26	16
*n*=*167*	II	65	39
	III	76	46
Size	Up to 2 cm	67	40
*n*=*165*	>2 to 5 cm	98	59
Nodal status	Negative	73	44
*n*=*167*	Positive	64	38
Lymphovascular invasion	Negative	95	57
	Positive	71	43
*n*=*166*			
PgR status	Negative	84	57
*n*=*147*	Positive	63	43
Ki67 status	Negative	72	51
*n*=*142*	Positive	70	49
ER*α* status	Negative	53	33
*n*=*160*	Positive	107	67

). All patients received adjuvant hormone therapy but no chemotherapy. For 143 cases, endocrine therapy consisted of adjuvant Tamoxifen only. Since steroid receptor analysis was not routinely performed until 1996, some cases were subsequently found to be ER*α*-negative. All cases were subjected to full histopathological review, by three investigators (PAO′N, CSF and JPS) according to the UK NHSBSP guidelines (National Coordinating Group for Breast Screening, 1997). Clinical follow-up data, with informed consent, were recorded by retrospective case-note review. Ethical approval for the study was obtained from all relevant bodies.

### Immunohistochemistry

Mouse anti-(human ER*β*1) monoclonal antibody PPG5/10 was employed to recognise the ER*β*1 isoform (Serotec Ltd, Kidlington, Oxford, UK). Specificity of the antibody has previously been confirmed by Western blotting in our laboratory (Shaaban *et al*, 2003b). For the immunohistochemical detection of ER*α*, a mouse anti-(human ER*α*) monoclonal antibody was used (Clone 1D5, Dako Ltd, Ely, Cambridge, UK). Progesterone receptor (PgR) status was measured using a mouse monoclonal anti-PgR antibody (Clone 1A6, Novacastra, Newcastle upon Tyne, UK). Ki67 status was assessed using polyclonal rabbit anti-human Ki67 antibody (Ki67p, Novacastra, Newcastle upon Tyne, UK).

Formalin-fixed and paraffin wax-embedded sections of normal, benign and malignant breast tissues were immunostained for ER*α* and ER*β*1. The methods were identical to those previously described (Shaaban *et al*, 2002), but with the addition of an overnight incubation at 4°C for the ER*β*1 antibody diluted (1 : 2) in Tris buffer (pH 7.2) containing 1% (w/v) BSA. Immunostaining for ER*α* was performed by incubating sections with the mouse anti-ER*α* monoclonal antibody for 40 min at room temperature. Positive and negative controls were included for each antibody and in each batch of staining.

Analysis was restricted to the epithelial component of all tissues. To maximise consistency of scoring, only nuclei having moderate or strong staining were regarded as positive, irrespective of cytoplasmic staining. The percentage of positively stained epithelial cells was calculated as a proportion of the total number of epithelial cells present. For ER*β*1, cases were considered as positive only when more than 20% of cells were stained, as previously described (Jarvinen *et al*, 2000; Miyoshi *et al*, 2001; Shaaban *et al*, 2003b), although other cutoff values were also tested. In contrast with ER*α*, there has been no agreement on the cutoff value for defining ER*β* positivity. We chose a cutoff value of 20% as previously described in the studies by Jarvinen *et al* (2000) and Miyoshi *et al* (2001), as well as to be consistent with our previous study (Jarvinen *et al*, 2000; Miyoshi *et al*, 2001; Shaaban *et al*, 2003b). A 10% cutoff (consistent with that employed in our previous studies) was applied as the conventional criterion to define positive ER*α* or PgR staining (Sannino and Shousha, 1994). Ki67 was regarded as elevated if >20% cells were stained, based on the median expression in this cohort of cases.

### Reverse transcription (RT)–PCR analysis

Total RNA (5 *μ*g) was provided by the CTBRC. Following DNAaseI digestion (Gibco), RT was performed in duplicate on 0.5 *μ*g of RNA, according to the manufacturers' instructions (Gibco). Reverse transcription reactions incorporated Superscript II Reverse Transcriptase (Gibco), 0.5 *μ*g Oligo (dT)_12−18_ and 0.5 *μ*l Prime Recombinant Ribonuclease Inhibitor (Eppendorf). Parallel reactions were performed in which the RT enzyme was omitted and these acted as controls for genomic DNA contamination. Polymerase chain reactions were performed in 20 *μ*l duplicate volumes in 96-well plates, each using 2 *μ*l of a 1/20 dilution of cDNA per reaction (equivalent to cDNA from approximately 2.5 ng of total RNA). All PCR reactions included 0.2 mM dNTPs, 0.5 U of HotstarTaq DNA polymerase (Qiagen) and 1 × PCR buffer (containing 1.5 mM MgCl_2_, Qiagen). Oligonucleotide primers for RT–PCR and the conditions used are shown in Table 2

**Table 2:** Primer sequences, conditions, and product sizes in base pairs (bp) for RT–PCR

**PCR**	**Primer sequence**	**[Primer] (*μ*M)**	**[MgCl_2_] (mM)**	**Size (bp)**
ER*α*	AGACATGAGAGCTGCCAACC	2	1.5	299
	GCCAGGCACATTCTAGAAGG			
				
ER*β*1	CCAAATGAGGGACCACACAGCAG	1	1.5	476
	AGTATGTACCTCTGGTCACAGCG			
				
*β*-actin	TGACGGGGTCACCCACACTGTGCCCATCTA	0.48	1.5	610
	CTAGAAGCATTTGCGGTGGACGATGGAGGG			
				
HPRT	CTATTGTAATGACCAGTCAACAGGGG	1	3	367
	AACTCAACTTGAACTCTCATCTT			

and have been previously validated (Moore *et al*, 1998; Kurebayashi *et al*, 2000). Primer concentrations and final MgCl_2_ concentrations varied according to Table 2. The PCR reaction used for ER*β* is specific for the ER*β*1 isoform (Moore *et al*, 1998). *β*-Actin and hypoxanthine ribosyltransferase (HPRT) were used as control genes to determine RNA integrity and RT efficiency. Care was taken to ensure that each PCR reaction was limited in cycle number, thus to avoid the plateau phase of the reaction. Oestrogen receptor *α* RNA was assessed both by ER*α* PCR and duplex PCR for ER*α* and actin primers (Kurebayashi *et al*, 2000). The data were highly concordant (Chi-square, *P*<0.001). Polymerase chain reactions for ER*β*, actin and HPRT were performed individually.

Positive controls using MCF-7 cell line cDNA for ER*α* and testis cDNA for ER*β*1 were included together with negative controls in each reaction plate. Polymerase chain reaction was performed using Perkin-Elmer 9600 thermal cyclers. All cycling reactions were preceded by a pre-incubation at 94°C for 13 min, and were followed within a 3 min final extension at 72°C. Cycling conditions for reactions are given in Table 3

**Table 3:** Conditions of sequence-specific PCR cycling reactions

		**Condition**		
**Oligonucleotide**	**Cycle**	**Denaturation phase**	**Annealing phase**	**Extension phase**
ER*α* and *β*-actin	35	94°C, 15 s	58°C, 15 s	72°C, 30 s
ER*β*1	40	94°C, 30 s	64°C, 40 s	72°C, 45 s
HPRT	36	94°C, 30 s	60°C, 60 s	—

.

Polymerase chain reaction products were separated by electrophoresis on gels containing 2.5% Seakem Agarose (Flowgen) and TAE buffer (40 mM Tris acetate, 1 mM EDTA, pH 7.6). Molecular weight markers (PhiX174/HaeIII, Abgene) were included on each gel and DNA was visualised by inclusion of 0.5 *μ*g ml^−1^ ethidium bromide, scanning with a Molecular Dynamics FluorimagerSI and analysis with ImageQuant version 4.1 software (Molecular Dynamics).

The identity of PCR products was confirmed by direct sequencing using DYEnamic ET Dye Terminator Cycle Sequencing Kit for MegaBACE (Amersham Pharmacia Biotech) and analysed on a MegaBACE 1000 (Molecular Dynamics). Alternatively, PCR products were cloned using TOPO-TA cloning (Invitrogen) prior to sequence analysis.

The presence of a PCR product was assessed independently by two investigators (PAO′N and MPAD) and scored as positive where both agreed. Control genes, actin and HPRT were scored as weak or strong positive and individual RT reactions excluded from ER assessment if either gene was negative, or if both were only weak. Cases were considered positive for ER*α* or ER*β* if any band was seen regardless of the intensity.

### Statistical analysis

All statistical analyses were performed using the SPSS® package (Windows, v.11). To compare the immunohistochemical percentage values for ER*α*, PgR, Ki67 or ER*β*1 in different groups, data were analysed by the nonparametric, two-sided Mann–Whitney test and the two-sided *T*-test. The nonparametric, two-sided Mann–Whitney test was also used for other ordinal data such as stage and grade. Association between categorical data was assessed by the Chi-squared test and correlations between interval data were tested using Pearson's correlation coefficient. Survival curves were generated using the Kaplan–Meier method for censored data and compared using the log-rank test. Cox's regression models were used for multivariate survival analysis.

## RESULTS

### RT–PCR

**Figure 1:**
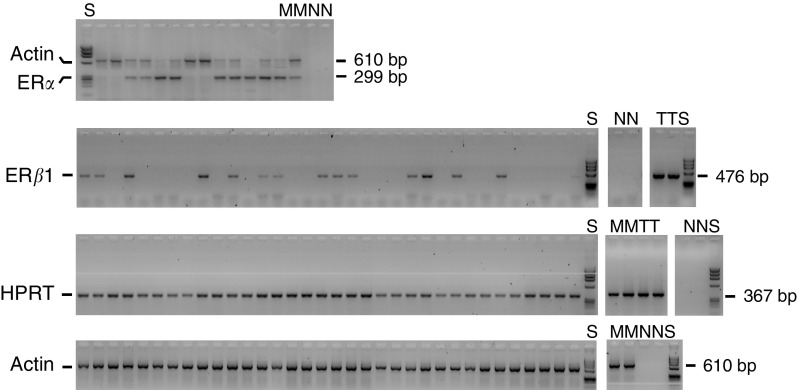
Reverse transcription–PCR analysis of ER*α*, ER*β*, actin and HPRT. Oestrogen receptor *α* and actin PCR were performed as a duplex (top) and all other PCRs as single reactions. Controls included were cDNA negative reactions (N), MCF-7 cDNA (M) and testis cDNA (T). All samples were run on agarose gels with *Phi*X174/*Hae*III DNA size markers (S).

The identities of representative RT–PCR products for each gene were confirmed by sequence analysis. No evidence of artefactual PCR products due to genomic DNA contamination was identified. An example of RT–PCR analysis is shown in Figure 1

. The use of control genes *β*-actin and HPRT identified 127 cases in which cDNA was considered to be of appropriate quantity and integrity for further analysis. The results of these two control genes were in agreement (Chi-square 27, *P*<10^−6^). Reverse transcription–PCR analysis categorised 66% cases as ER*α*-positive and 68% cases as ER*β*1-positive (Table 4

**Table 4:** Relationships between ER*α* and ER*β*1 as assessed by immunohistochemistry (IHC) and RT–PCR

		**ER*α* IHC**		
**A**		**neg.**	**pos.**	**Chi-square (P)**
ER*β*1 IHC	neg.	4	16	1.6 (0.21)
	pos.	40	77	

		**ER*α* RT–PCR**		
**B**		**neg.**	**pos.**	**Chi-square (P)**
ER*α* IHC	neg.	33	12	47 (<0.0005)
	pos.	9	67	
ER*β*1RT-PCR	neg.	23	18	12 (<0.0005)
	pos.	21	65	
ER*β*1 IHC	neg.	3	8	0.23 (0.64)
	pos.	31	59	

		**ER*β*1RT–PCR**		
**C**		**neg.**	**pos.**	**Chi-square (P)**
ER*α* status	neg.	20	25	4.9 (0.027)
	pos.	19	57	
ER*β*1 IHC	neg.	4	7	0.08 (0.78)
	pos.	29	61	

). In all, 51% were positive for both ER*α* and ER*β*1, 18% negative for both, 17% positive only for ER*α* and 14% positive only for ER*β*1. There was some association between RT–PCR results for each ER (Chi-square 12.3, *P*<0.0005). The distribution of ER*β*-positive tumours was significantly different between ER*α*-positive and -negative cases.

### Immunohistochemistry

Immunostaining for ER*α* was performed on 149 cases and was nuclear in all cases regarded as positive. Cytoplasmic-only staining was excluded. Cytoplasmic staining for ER*β* has been described in several studies and is likely to be genuine and not a staining artefact, although the precise significance of cytoplasmic staining remains unknown. Similar to ER*α*, cytoplasmic staining without nuclear expression was considered negative, so that only nuclear expression was interpreted as positive to maintain convention and comparability with previously reported studies. Using a cutoff value of 10%, 49 cases (33%) were ER*α*-negative by immunohistochemistry and the remaining 100 cases (67%) classed as ER*α*-positive. Oestrogen receptor *α* status was available for a further 11 cases by case-note review (Table 1). Conventionally, the epithelial component only was scored on assessing ER*α* and ER*β* positivity. Oestrogen receptor *α* was expressed in the epithelial cells, but ER*β* was also expressed in the stroma.

**Figure 2:**
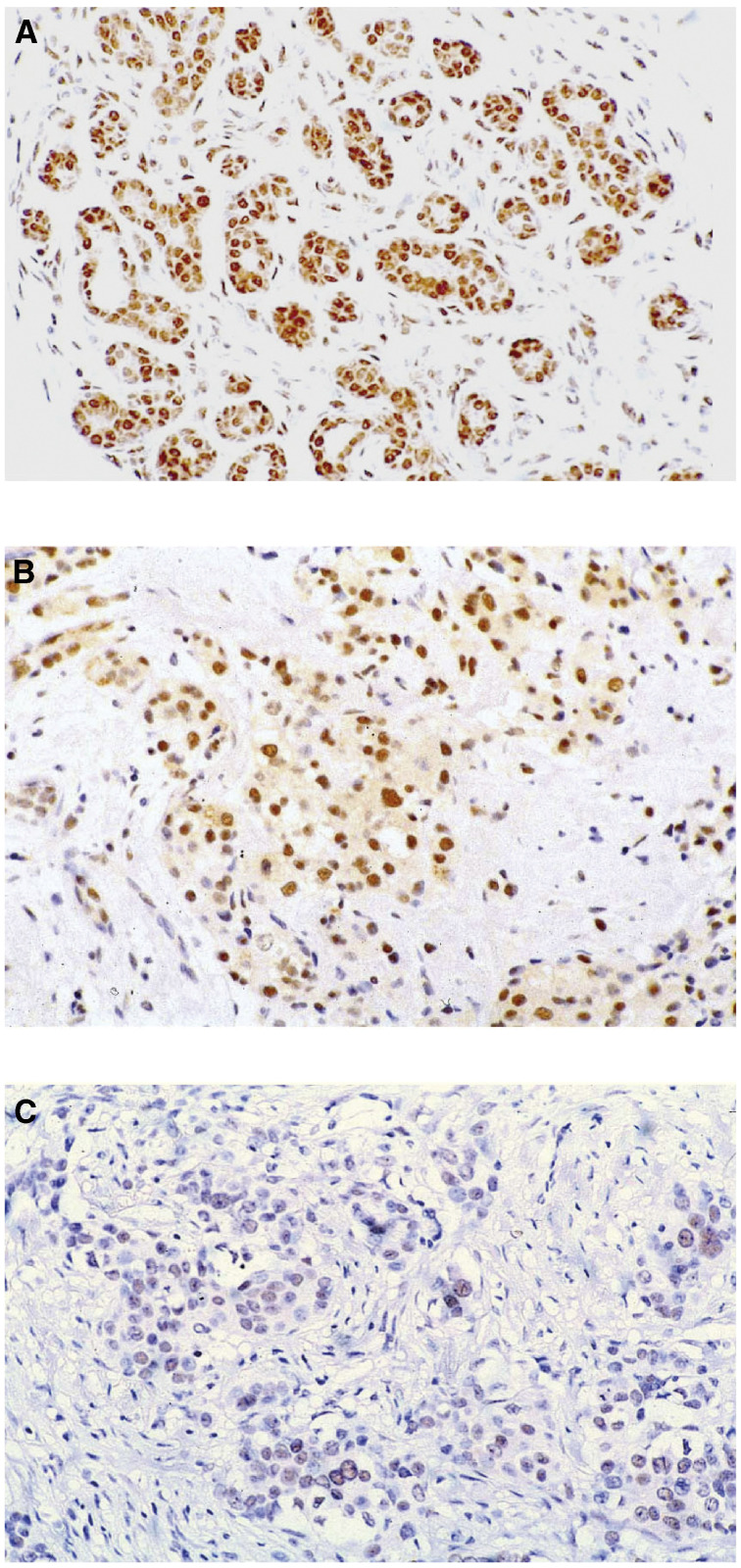
Immunohistochemical staining with ER*β*1 antibody. In normal breast (**A**), a strong nuclear staining of the majority of luminal cells is seen and the myopeithelial cells and stromal cells also express the protein. Examples of invasive ductal carcinoma of no special type show either strong nuclear expression with some cytoplasmic staining (**B**) or staining of only a few positive cells (**C**).

**Figure 3:**
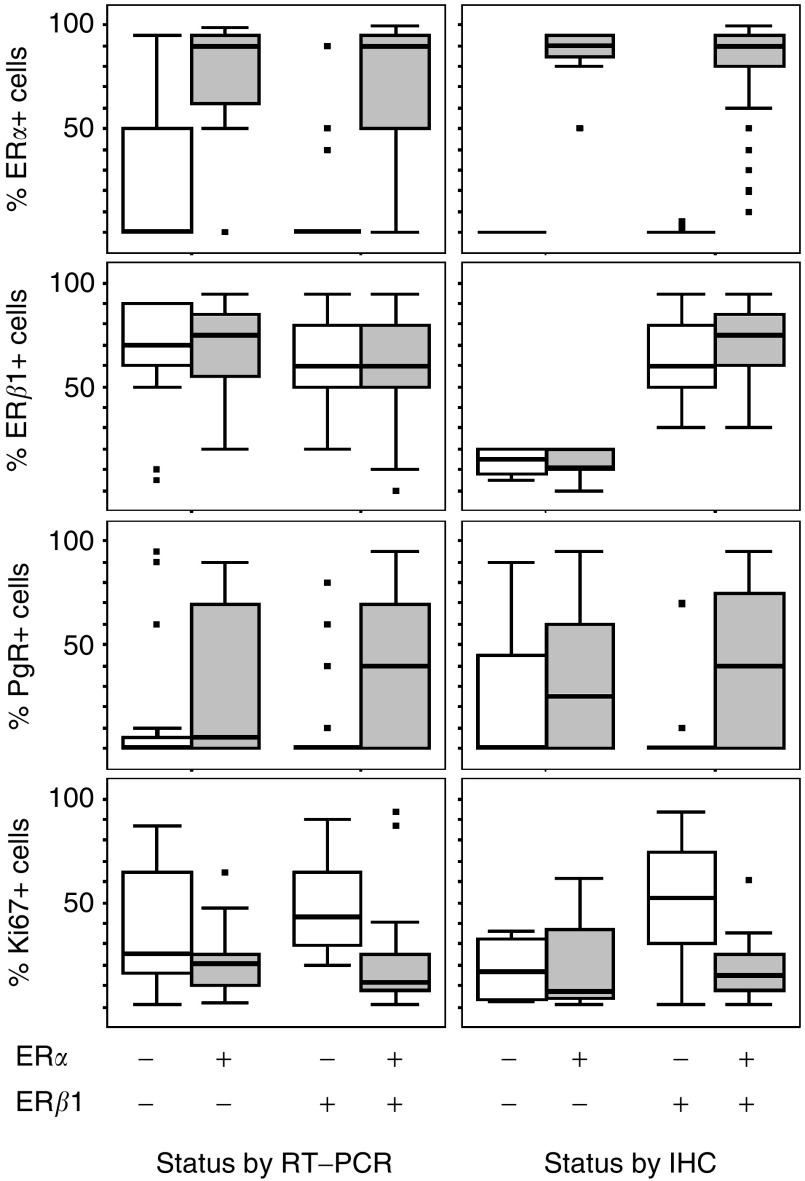
Boxplots of immunohistochemistry data (% immunopositive cells) for ER*α*, ER*β*1, PgR and Ki67 categorised by RT–PCR results (left) or IHC results (right) for ER*α* and ER*β*1. The box represents the interquartile range, the line across the box indicates the median and the whiskers extend from the box to the highest and lowest values (excluding outliers and extreme points). Square, black markers represent outliers and extreme points.

Immunostaining for ER*β*1 was performed on 138 cases. Epithelial cells were considered positive if nuclear staining was identified (Figure 2

). Cytoplasmic staining co-existent with nuclear staining was identified in 64 cases (Figure 2). In contrast with ER*α*, there has been no agreement on the cutoff value for defining ER*β* positivity. The conventional cutoff value for ER*α* positivity is 10% (Sannino and Shousha, 1994). We used a cutoff value of 20% as previously used in the studied by Jarvinen *et al* (2000) and Miyoshi *et al* (2001), as well as to be consistent with our previous study (Shaaban *et al*, 2003b). The 20% cutoff (Jarvinen *et al*, 2000; Miyoshi *et al*, 2001; Shaaban *et al*, 2003b) for ER*β*1 staining resulted in a high proportion of positive cases (85%, 118 cases). The mean percentage of stained cells was 14% for negative cases and 69% for positive cases (*T*-test, *P*<0.0005). The proportion of immunostained cancer cells was considerably more variable for ER*β*1 than for ER*α* (Figure 3

).

Immunohistochemical data for both ER*α* and ER*β*1 were available for 137 cases. In all, 56% of all cases were positive for both ERs (Table 4) and only 3% were negative for both ER*α* and ER*β*1. However, a significant proportion (29%) was negative for ER*α* but expressed ER*β*1, compared to a smaller number of cases expressing ER*α* alone (12%). There was no significant association between ER*α* and ER*β*1 status (Chi-square 1.6, *P*=0.21). No correlation between the proportion of immunopositive cells was identified (Pearson's *R*=−0.007, *P*=0.94).

### Relationship between ER RT–PCR and immunohistochemistry

Reverse transcription–PCR for ER*α* was performed on 121 cases with known ER*α* immunohistochemistry status. There was a significant association between the two techniques (Chi-square 47, *P*<0.0005). For ER*α* RT–PCR-negative cases, the median expression of ER*α* by immunohistochemistry was zero. For ER*α* RT–PCR-positive cases, median expression of ER*α* by immunohistochemistry was 90% (Figure 3). The proportion of immunostained cells was significantly higher in cases positive for ER*α* by RT–PCR (both *T*-test and Mann–Whitney *P*<0.0005).

Both RT–PCR and immunohistochemistry were assessed for ER*β*1 in 101 cases (Table 4). No significant relationship was identified between RT–PCR and immunohistochemistry (Chi-square 0.8, *P*=0.78). This was true for all ER*β*1 immunohistochemical cutoffs tested. There was no significant association between ER*α* RT–PCR and ER*β*1 immunohistochemistry (Chi-square 0.23, *P*=0.64); although there was an association between ER*β*1 RT–PCR and positive ER*α* immunohistochemical status (Chi-square 4.0, *P*=0.027), the mean ER*α* scores were not significantly different in ER*β*1 RT–PCR-negative and -positive cases (45 and 55%, respectively, *T*-test *P*=0.25).

### Relationship between ER immunohistochemistry and other parameters

Oestrogen receptor *α* expression, determined by immunohistochemistry, was associated with PgR status and Ki67 status (Table 5

**Table 5:** Relationship between ER*α*, ER*β* and other histopathological variables

**Factor**	**ER*α* IHC**	**ER*α* RT–PCR**	**ER*β*IHC**	**ER*β* RT–PCR**
Stage	*low* *P*=0.019	*low* *P*=0.014	n.s.	n.s.
	(MW) *n*=160	(MW) *n*=127	(CS, MW) *n*=138	(CS, MW) *n*=127
Grade	*low P*<0.0005	*low P*<0.0005	n.s.	n.s.
	(MW) *n*=160	(MW) *n*=127	(CS, MW) *n*=138	(CS, MW) *n*=127
Size	n.s.	n.s.	*larger* *P*=0.076*	*larger* *P*=0.025
	(CS, MW) *n*=158	(CS, MW) *n*=125	(MW) *n*=136	(MW) *n*=125
Nodal status	*negative* *P*=0.048	*negative* *P*=0.057	n.s.	n.s.
	(CS) *n*=131	(CS) *n*=108	(CS) *n*=110	(CS) *n*=108
Lymphovascular Invasion	n.s.	n.s.	*present* *P*=0.067*	*present* *P*=0.043
	(CS, MW) *n*=159	(CS) *n*=126	(CS) *n*=137	(CS) *n*=126
PgR	*positive P*<0.0005	*positive P*<0.0005	n.s.	*positive P*=0.056
	(MW) *n*=146	(CS) *n*=110	(CS, MW) *n*=138	(CS) *n*=110
Ki67	*low* *P*<0.0005	*low* *P*<0.0005	*high* *P*=0.034	n.s.
	(MW) *n*=141	(CS) *n*=108	(MW) *n*=133	(CS, MW) *n*=108

Statistical test used was chi-square (CS), Mann-Whitney (MW); n.s.=not significant.

* using a cut-off of 60% positive cells for ER*β*1.

). Mean PgR staining was significantly higher in ER*α*-positive cases (37 *vs* 4.7%, *T*-test *P*<0.0005), while the mean Ki67 was significantly lower (16 *vs* 47%, *T*-test *P*<0.0005). Positive ER*α* status was also associated with low-stage, low morphological grade and negative nodal status. No association between ER*α* status and tumour size or lymphovascular invasion was revealed.

No significant association was detected between ER*β*1 immunohistochemical expression and grade of tumour, axillary nodal status, ER*α* status or PgR status. There was an association with greater proliferation, measured by Ki67 staining. The mean % Ki67-positive cells was greater (*T*-test *P*=0.043) in the ER*β*1-positive cases (28%) than in the ER*β*1-negative cases (18%). This was true even when considering ER*α*-negative cases, but not ER*α*-positive cases; Ki67 is greater (*T*-test *P*=0.022, Mann–Whitney *P*=0.028) in ER*β*1-positive/ER*α*-negative cases (mean 51%) than in ER*β*1-negative/ER*α*-negative cases (mean 18%). Using a median cutoff of 60% (but not a cutoff at 20%), there was a trend for the presence of lymphovascular invasion and larger tumours in ER*β*1-positive cases, as seen for RT–PCR.

### Relationship between ER RT–PCR and other parameters

**Figure 4:**
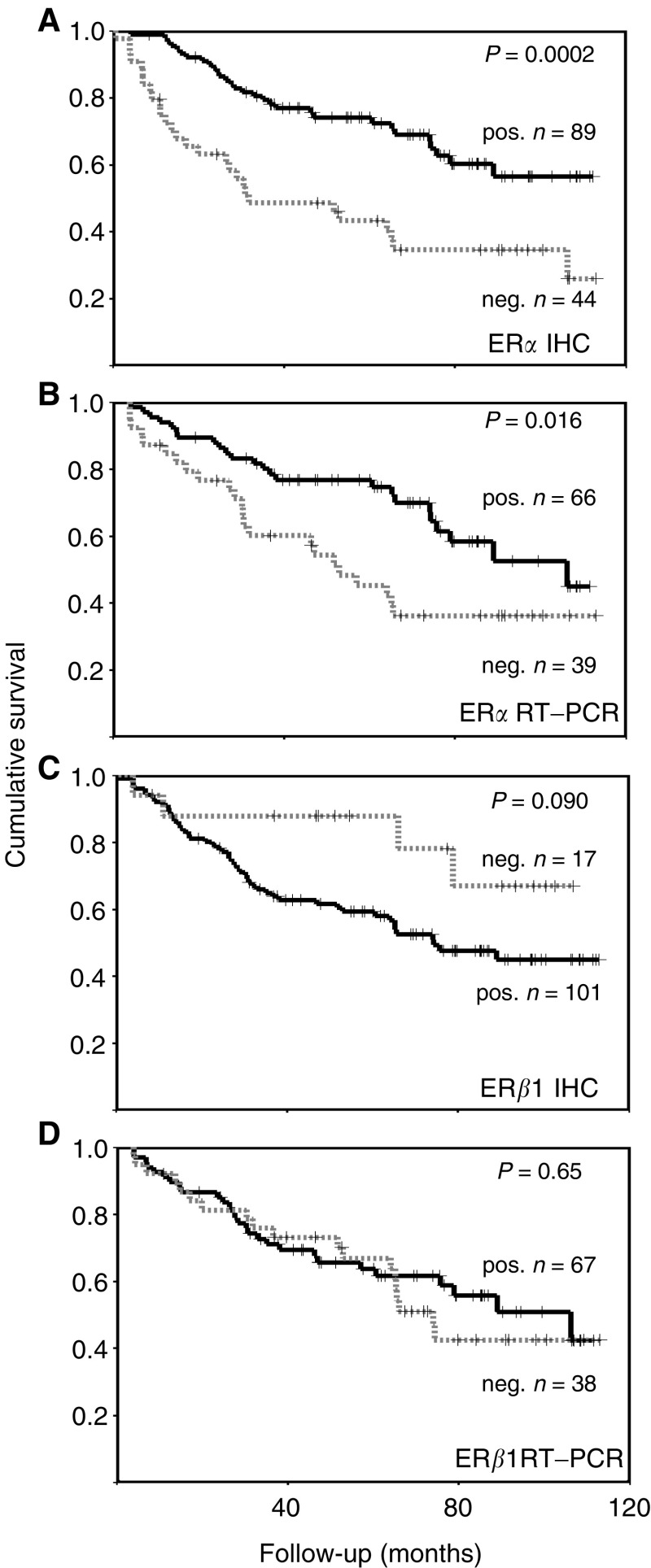
Kaplan–Meier RFS curves for ER*α* status (**A**), ER*α* RT–PCR (**B**), ER*β*1 IHC (**C**) and ER*β*1 RT–PCR (**D**). Dotted lines are negative cases and unbroken lines positive cases, crosses represent censored data, *P-*values are given for log-rank tests.

While there was no relationship between ER*β*1 RT–PCR status and Ki67 staining and only a trend for an association with PgR, there was an association (Figure 4

, Table 5) between these parameters and ER*α* RT–PCR status. Oestrogen receptor *α* RT–PCR-positive status was associated with a significantly higher mean PgR staining (36% compared to 13% in negative cases, *T*-test *P*<0.001, *n*=110) and lower mean Ki67 (19% compared to 41% in negative cases, *T*-test *P*<0.0005, *n*=109). The relationships between ER*α* RT–PCR and either PgR or Ki67 remain largely unchanged (Figure 3), irrespective of ER*β*1 RT–PCR status, although Ki67 levels are somewhat higher in ER*β*1-positive/ER*α*-negative cases. As expected from their ER*α* status, such tumours had significantly higher Ki67 values and lower PgR values than either ER*α*-positive subgroup (*T*-test *P*<0.04, Mann–Whitney *P*<0.007), but values for these markers were not significantly different from the ER*β*1-negative/ER*α*-negative subgroup (*T*-test and Mann–Whitney *P*>0.08).

Demonstration of ER*α* expression by RT–PCR was significantly associated with low stage and low grade. There was a trend with nodal status, but no association with size or lymphovascular invasion. No relationship between ER*β*1 RT–PCR and stage, grade or nodal status was identified, although associations with lymphovascular invasion and larger tumours were detected (Table 5).

Cases that were ER*β*1-positive/ER*α*-negative by RT–PCR had larger tumours (mean 4.1 cm) than those that were ER*β*1-positive/ER*α*-positive (2.7 cm, *T*-test *P*<0.0005), ER*β*1-negative/ER*α*-negative (2.2 cm, *T*-test *P*=0.001) or ER*β*1-negative/ER*α*-positive (3.1 cm, Mann–Whitney *P*=0.012). These ER*β*1-positive/ER*α*-negative tumours were also of higher stage than the other three subgroups defined by RT–PCR (Mann–Whitney *P*<0.017).

### Relationship between ER and disease outcome

In order to examine the possible effect of ER*β*1 status in a cohort of patients receiving the same endocrine treatment, outcome data have been restricted to those 143 women receiving adjuvant Tamoxifen, but without primary endocrine treatment and no primary or adjuvant chemotherapy. In this cohort, ER*α* expression determined by immunohistochemistry (Figure 4), stage, grade, size, nodal status, Ki67 staining and PgR were all associated with the expected manner that measures of breast cancer relapse-free survival (RFS) and demonstrated significant differences in breast cancer-associated survival (BCS) and overall survival (OS). All these markers had significant log-rank scores for RFS, BCS and OS (all log-rank *P*<0.021). Lymphovascular invasion only exhibited a trend for poorer outcome (*P*=0.08 for RFS, *P*=0.09 for BCS). In multivariate analysis, ER*α* immunohistochemistry status was independently significant for RFS in the presence of each other parameter apart from grade and Ki67 status. Considering multiple parameters, the strongest significance was attached to nodal status, followed by grade.

Positive ER*α* immunohistochemical scores were associated with better RFS using all cutoff points from the standard 10–90% positive cells (*P*<0.001). Positive ER*α* RT–PCR status was associated with a better outcome, as measured by RFS (Figure 4), but not BCS (*P*=0.055) or OS (*P*=0.21). No significant association with outcome was seen for ER*β*1 RT–PCR (Figure 4; *P*=0.65 RFS, *P*=0.27 BCS, *P*=0.87 OS). There was a trend for better survival in cases immunohistochemically negative for ER*β*1 (Figure 4C). Only four of the 17 (24%) ER*β*1-negative cases relapsed, when compared to 51 of 103 (50%) ER*β*1-positive cases (Fisher's exact test, *P*=0.029). This was not true for any other cutoff tested.

Within the adjuvant Tamoxifen cohort, 91 cases were ER*α*-positive by immunohistochemistry and therefore typical of those women who are likely to receive adjuvant endocrine treatment today. Within this subgroup grade, nodal status and Ki67 were all significant markers of outcome (RFS, BCS and OS all log-rank *P*<0.01), as were stage (RFS log-rank *P*<0.01, BCS *P*=0.03), PgR (BCS, RFS and OS all log-rank *P*<0.05) and size (OS log-rank *P*=0.015). ER*β*1 RT–PCR showed no association with any measure of outcome, but as before a trend for a worse outcome in ER*β*1 immunohistochemically positive cases was seen (RFS, log-rank *P*=0.11; Fisher's exact test, *P*=0.061).

## DISCUSSION

This study has confirmed the initial hypothesis that comparison of protein expression levels for ER*α* and ER*β*1, together with their respective mRNA levels, are indicators of the clinical and pathological characteristics of a cohort of postmenopausal primary breast cancer patients treated only surgically and thereafter with Tamoxifen therapy. The findings confirm the differential expression of these two oestrogen receptors by human breast carcinomas, with a high degree of correlation between the immunohistochemical and RT–PCR data for ER*α* being identified.

Unlike the findings for ER*α*, this study did not reveal a strong correlation between ER*β* RT–PCR and the corresponding immunohistochemistry. Identification of the technical and biological reasons for this apparent discrepancy is of fundamental importance to understanding the role of ER*β* in human breast cancer. Recently, Omoto *et al* (2002) reported that protein and RNA levels are often not in agreement, but did not provide any cogent explanation for this discrepancy. Three potentially important factors require consideration: First, this apparent discrepancy might be explained by relative lack of sensitivity of RT–PCR when compared to the 20% immunohistochemical cutoff (where 29% of cases were RT–PCR negative and immunohistochemically positive). Previously, it has been reported that mRNA levels for ER*β* are lower and more diverse than those for ER*α* (Iwao *et al*, 2000) and that levels of ER*β*1 are lower than for other ER*β* variants (Leygue *et al*, 1999; Iwao *et al*, 2000). Either of these phenomena would contribute to a lower sensitivity for detection of ER*β*1 by RT–PCR. When testing for a possible correlation between ER*β*1 identified by RT–PCR and by immunohistochemistry, various cutoff levels were assessed. However, there was no value that gave a statistically significant association with the RT–PCR data. In contrast, correlations occurred between ER*β*1 RT–PCR and with both ER*α* immunohistochemistry and RT–PCR, which were not recapitulated at the protein level and which would account for other reports of relationships between the two ERs. Second, while immunohistochemistry is an *in situ* technique in which data are obtained subjectively, RT–PCR is performed on disaggregated tissue preparations in a quantitative manner. Hence, expression of ER*β*1 mRNA from other cell types might account for the seven cases that were RT–PCR positive and immunohistochemically negative, but not the 29 RT–PCR-negative but immunohistochemically positive cases. While a theoretical possibility, this explanation is interesting since nonepithelial stromal cells of normal breast tissues have been found to be weakly ER*β*-positive while stromal cells of the unusual phylloides tumours were found to strongly express ER*β* (Shaaban *et al*, 2003b).

Translational or post-translational control mechanisms are likely to play a significant role in ER*β* expression in some cases of breast cancer. We have already shown that modulation of ERs is both complex and indirect, the latter mechanisms including altered expression of homeostatic protein hsp-27 (O'Neill *et al*, 2003). It is now recognised that the precise structure of many proteins expressed by individual genes varies with the phenotypic status of an individual cell. These ‘splice variants’, while encoded within the normal genome, become expressed according to the overall status of the tissue in which they originate (e.g. embryonic, adult-proliferative or malignant). Such differences are already recognised to be important, with respect to splice variants of some proteins (e.g. voltage-gated ion channels), but are yet to be proven for others (e.g. ERs), although there is substantial circumstantial evidence for this selection. If splice variation is an important factor in the expression of ER*β*, then use of monoclonal antibodies directed to epitopes in the wild type that become spliced out and hence nonexpressed in the cancers provide erroneous information. Recognition of this caveat is important for accurate interpretation of such data. Multiple forms of ER*β* splice variants occur in normal breast tissue and breast malignancies (Leygue *et al*, 1999; Omoto *et al*, 2002). Unfortunately, most previous RT–PCR analysis studies have used primers unsuitable for distinguishing individual isoforms. Recent production of antibodies suitable for detection of individual ER*β* isoforms (Saunders *et al*, 2002; Skliris *et al*, 2002) should allow a better understanding of the complex factors regulating hormone responsiveness of human breast carcinomas to emerge (O'Neill *et al*, 2003; Shaaban *et al*, 2003b).

In the current series, and in accordance with previous immunohistochemical reports (Enmark *et al*, 1997), ER*β*1 was predominantly localised to the nuclei of epithelial cells and of myoepithelial cells, as well as stromal cells (Taylor and Al-Azzawi, 2000; Speirs *et al*, 2002). Oestrogen receptor *β*1 expression was identified in 85% of invasive cancers using a 20% immunohistochemical cutoff and the median expression was 60%. The reported proportion of ER*β*1-positive invasive carcinomas varies appreciably among previous studies and might be explained by differences in the specificity of the antibodies, methods of antigen retrieval and different thresholds used to define positive staining (Skliris *et al*, 2002; Shaaban *et al*, 2003a). In the current cohort, 56% of cancers were positive for both ER*α* and ER*β*1 by immunohistochemistry, while 29% of cancers were ER*α*-negative and ER*β*1-positive. Given the potential discrepancies due to antibody usage and staining technique, together with the robust levels of ER*β*1 staining, these numbers are in agreement with those reported in other immunohistochemical studies of ER*β*1 (Jarvinen *et al*, 2000; Omoto *et al*, 2002; Saunders *et al*, 2002), which describe 48–74% of cases as ER*β*1-positive/ER*α*-positive and 8–20% as ER*β*1-positive/ER*α*-negative. Unlike ER*α*, which is usually expressed in only a minority of cells in normal epithelium and aberrantly expressed at high levels in the majority of cells in many breast cancers, ER*β*1 is apparently expressed in the majority of cells in normal breast and this expression is maintained in most breast cancers at a variety of levels. Persisting but varied expression of ER*β*1 in the presence or absence of greater amounts of ER*α* indicates that the role played by the interaction between ER*α* and ER*β*1 during mammary carcinogenesis and in subsequent cancers is likely to be complex. Thus, this study has pinpointed a cellular control mechanism that, in human breast cancer, requires specific and detailed analysis.

Only one previous study has reported ER*β* immunohistochemistry in adjuvant Tamoxifen-treated patients (Mann *et al*, 2001). In contrast to our present findings, the previous adjuvant study suggested ER*β*-positive patients to have a better survival when compared with ER*β*-negative patients. Overall, the immunostaining reported in the previous study appeared weaker than observed here, with only 66% of 118 cases being ER*β*-positive at a 10% cutoff, when compared to 85% positive for ER*β*1 at a 20% cutoff. Since that study utilised an antibody with broad specificity, possible contribution of other ER*β* variants is unclear. It is possible that the findings of that study are due to expression of variants ER*β*2 and ER*β*5 since, at the RNA level, these have been shown to be greater than ER*β*1 in breast cancers (Leygue *et al*, 1999; Omoto *et al*, 2002). The isoform of ER*β* to have the greatest effect on outcome for breast cancer patients is yet to be confirmed. Although only seen here for RT–PCR, others have reported some association between ER*α* and ER*β* staining (Jarvinen *et al*, 2000; Omoto *et al*, 2001) and it is possible that the unreported ER*α* status of the cases previously reported has some influence on the data (Mann *et al*, 2001). In this study, no association was found between ER*β* and grade of tumour, progesterone receptor, or nodal status, thus broadly in agreement with other studies. However, in this cohort of post-menopausal women treated with Tamoxifen therapy, ER*β*-positive cancers tended to have poorer RFS than ER*β*-negative cancers. This finding was not entirely due to the presence of ER*β* in some ER*α*-negative cases, since a trend was still present in the ER*α*-positive subgroup.

This study lends some support to the original hypothesis that expression of wild-type ER*α* influences the effectiveness of antioestrogen therapy. Furthermore, antioestrogens (e.g. Tamoxifen) of particular affinity for the specific splice variant of oestrogen receptor expressed by each individual breast carcinoma may have agonistic effects in ER*β*-positive tumours, hence resulting in a lack of efficacy of hormonal therapy (Speirs *et al*, 1999). There is evidence that this might also be true for ER*β*2, since this protein was associated with poor response to Tamoxifen in a neoadjuvant setting (Saji *et al*, 2002). However, our data contradict less critical reports that are imprecise with respect to patient groups examined and ER*β* variants detected. Further studies are now being performed to clarify the roles of different ER*β* splice variants in breast cancers treated by hormonal manipulation. These will include cohorts of patients selected according to clinical and treatment criteria in order to determine the importance of ER*β* in breast cancer management and outcome.
